# Investigating spiritual care perceptions and religious coping methods among the relatives of terminally ill patients during the COVID-19 pandemic: the case of Turkey

**DOI:** 10.1186/s12904-024-01463-x

**Published:** 2024-05-22

**Authors:** Zuhal Cetın, Betul Ozen

**Affiliations:** 1 Health Sciences Institute, Erciyes University, 38033 Kayseri, Turkey; 2 Faculty of Health Sciences, Erciyes University, 38033 Kayseri, Turkey

**Keywords:** Terminal illness, Spirituality, Religiosity, Death

## Abstract

**Background:**

The COVID-19 pandemic in Turkey and around the world has had a profound impact on the families of terminally ill patients. In this challenging period, investigating the spiritual care perceptions and religious coping methods of patients' relatives is an essential step towards understanding the experiences in this process with the additional challenges brought by the pandemic and developing appropriate support services. This study aims to determine the spiritual care perceptions and the use of religious coping methods among the relatives of terminally ill patients in Turkey during the COVID-19 pandemic.

**Methods:**

The sample of this descriptive and correlational study consisted of the relatives of terminally ill patients (*n* = 147) who were receiving treatment in the Anesthesiology and Reanimation Intensive Care Unit of a state hospital in Turkey. Spirituality and Spiritual Care Rating Scale and the Religious Coping Scale to them using face-to-face interviews. Mann–Whitney U test, Kruskal–Wallis test, and Spearman's correlation analysis were used to analyze the data.

**Results:**

The mean age of the participants was 38.84 ± 11.19 years. Also, 63.3% of them were employed. The participant's total score on the Spirituality and Spiritual Care Rating Scale was 57.16 ± 6.41, and it was determined that the participants' level of perception of spirituality and spiritual care concepts was close to good. When the Religious Coping Scale scores of the participants were examined, it was found that both Positive Religious Coping levels (23.11 ± 2.34) and Negative Religious Coping levels (9.48 ± 1.47) were close to high. There was no correlation between the scores of RCOPE and SSCRS (*p* > 0.05).

**Conclusion:**

As a result, it was determined that the level of perception of spirituality and spiritual care concepts of the relatives of terminally ill patients during the COVID-19 pandemic was close to sound, and their Positive Religious Coping levels were high. Epidemics are a reality of the world, and it is essential to learn lessons from this process and take precautions for the future. We offer a perspective to realize the coping power of religion and spirituality, which are integral parts of life. The needs of terminally ill patients' relatives, a sensitive group, become visible.

## Background

Terminal illness inflicts agony upon patients and their relatives, evoking feelings of isolation, helplessness, anxiety, and the looming specter of death. This challenging period affects the patients, their families, and healthcare professionals [1, 2]. The burden is twofold: while patients contemplate their existence, their loved ones grapple with a sense of loss and uncertainty. However, with the support of caregivers and family members, the quality of life for terminally ill patients can be significantly improved. Caregivers should tailor their support to align with the patient's needs and family values [3].

Involvement of family members in patient care not only enhances the quality of care but also helps alleviate some of the challenges they face in maintaining their own lives. The fear of losing their loved ones prompts relatives to strive for their patients' comfort and continuity of care, avoiding isolation and interruptions in treatment [4]. However, relatives find solace in spirituality and religion by using spiritual coping strategies in this challenging process [5, 6].

Spirituality and religion, though distinct concepts, often intertwine in coping strategies during difficult times [7]. While spirituality emphasizes personal meaning and identity, religion provides a structured belief system. Both concepts complement each other, offering solace and guiding individuals through hardship [8]. Religious coping has been shown to impact mental well-being and resilience in adversity directly [9, 10].

The challenges faced by relatives of terminally ill patients have increased in intensive care units, especially during the COVID-19 pandemic. Isolation, visitor restrictions, and fear of death have exacerbated existing difficulties by preventing communication and access to religious rituals. [11]. The pandemic has created an environment where both religiosity and spirituality can play a vital role. The literature shows that healthcare professionals, patients, and some communities have conducted studies on spirituality and religious coping during the pandemic [12–17]. However, there are not enough studies on the relatives of patients negatively affected by this process. It is precious to raise awareness and make visible the needs and requirements of the relatives of terminally ill patients. Being aware of the importance of spirituality and religion in coping mechanisms, it is imperative to address the needs of patient relatives more effectively [18, 19]. Nurses are vital in reassuring patient relatives about their loved ones' well-being, even in their absence. Nurses should recognize the values and beliefs of the families they support and acknowledge the importance of spirituality and religion in their lives [20].

As a result, providing adequate support and care to relatives is crucial as they navigate the complexities of terminal illness with their loved ones. It is known that the difficulties and needs of the relatives of terminally ill patients have increased compared to previous periods, especially during the COVID-19 pandemic. The support and awareness to be provided in this challenging process are very important. It is necessary to draw attention to the difficulties experienced by the relatives of terminally ill patients by raising awareness about the importance of coping with the pandemic and similar difficulties, understanding spiritual and religious coping strategies, providing support, and taking measures. Healthcare professionals can provide a more compassionate and practical approach to support families on this challenging journey by understanding their relatives' needs, spiritual care perceptions, and religious coping methods. Thus, the quality of healthcare services can be improved, and a contribution can be made to improving the experiences of terminally ill patients and their relatives. Support and care services for the relatives of terminally ill patients need to be strengthened considering the challenging periods in the future. Therefore, this study aims to fill the gap in the field by raising awareness.

## Research questions

- Q1: What are the spiritual care perceptions of the relatives of terminally ill patients?
- Q2: What are the religious coping methods used by the relatives of terminally ill patients?
- Q3: Is there any correlation between spiritual care and religious coping methods in families of terminally ill patients?
- Q4: Do the scores of the Spirituality, Spiritual Care, and Religious Coping Scales vary according to exposure to COVID-19?

## Methods

### Research type

This research was a descriptive and correlational study.

### Sample and setting

The sample of this study consisted of the relatives of terminally ill patients who were receiving treatment in the Anesthesiology and Reanimation Intensive Care Unit of a state hospital in Turkey. This hospital is a regional hospital, and patients can come from all over the region. The Anaesthesiology and Reanimation Intensive Care Unit is a tertiary intensive care unit with 12 beds closed to visitors. Adult and elderly patients requiring intensive care are treated in this unit. The majority of patients are terminally ill. The Anesthesiology and Reanimation Intensive Care Unit has a Patient Relative interview room, and interviews with the patient's relatives are conducted there. The patient relatives' interview room is a quiet, socially distanced environment suitable for individual interviews with patient relatives.

The study's sample size was calculated as 147 individuals in G power version 3.1.9.7 at an effect size of 0.3, a confidence interval of 95%, and α 0.05. The sample consisted of 147 patient relatives who met the inclusion criteria and agreed to participate.

### Participants and data collection

The relatives of terminally ill patients to be included in the sample were determined as follows: First, the Anesthesiology and Reanimation Intensive Care Unit physician determined the diagnosis of the patient and the course of the disease, medical tests, imaging results, how severe the patient's condition is in line with expert opinions. At the end of this evaluation, the physician shared the information about the terminally ill patient with the nurse working in the Anaesthesiology and Reanimation Intensive Care Unit (this nurse is also the researcher of this study). The researcher communicated with the relatives of the terminally ill patient via telephone. Patients aged 18 years and over who were literate and could communicate effectively were informed about the purpose of the study. The researcher gave an appointment for a face-to-face interview to the relatives who agreed to participate in the study (inclusion/exclusion criteria were determined with the support of an intensive care specialist physician, an intensive care specialist nurse, and two faculty members who are experts in their field). Patient relatives were invited to the Patient Relative interview room belonging to the Anaesthesiology and Reanimation Intensive Care Unit, which was in line with the measures taken due to pandemic restrictions (mask, distance, hygiene, etc.). The patient relatives' interview room is a quiet, socially distanced environment suitable for individual interviews with patient relatives. The researcher and the patient's relative met in the patient's relative interview room on the specified day and time. The researcher explained the study's purpose to the terminally ill patients' relatives and obtained written and verbal consent. Then, the researcher applied the "Introductory Information Form," "Spirituality and Spiritual Care Rating Scale," and "Religious Coping Scale" using the face-to-face interview method. Data collection was completed in an average of 25 min. The researcher re-contacted the relatives of the patients who could not come to the appointment (due to COVID-19 disease and quarantine) and completed the data collection phase by giving another appointment at a suitable time interval.

#### Inclusion criteria

The inclusion criteria for the first and second-degree relatives of terminally ill patients were determined as follows:

- being 18 years old and over;
- being literate.
- being able to communicate effectively.
- having a family member confirmed by a physician to be terminally ill.

#### Exclusion criteria

The relatives of patients whom a physician did not confirm as terminally ill were excluded from the study.

### Data collection tools

#### Introductory ınformation form

This form consists of 11 questions about the socio-demographic characteristics of the patients and their relatives and their health-related characteristics.

#### Spirituality and Spiritual Care Rating Scale (SSCRS)

This scale was developed by McSherry, Draper, and Kendric in 2002 to assess the respondents' level of perceiving spirituality and spiritual care notions [21]. Ergül and Bayık-Temel conducted a Turkish validity and reliability study in 2007 [22]. This five-point Likert-type scale has 17 items and three subscales: Spirituality and Spiritual Care (SSC), religiosity (R), and personalized care (PC). The items are rated based on the responses ranging from [1] "Strongly Disagree" to [5] "Strongly Agree."Four items (3, 4, 13, and 16) of the scale are reversely scored. The minimum and maximum scores of the scale are 17 and 85, respectively. The scale has no cut-off point. A high score indicates that the respondent has a high level of perception on spirituality and spiritual care concepts (e.g., "I think spirituality is about having hope for life," "I think spirituality is a unifying force that enables people to be at peace with themselves and their environment"). The Cronbach's alpha coefficient of the scale is 0.64. It is considered an acceptable value for reliability since it is a novel assessment tool. In this study, Cronbach's alpha reliability coefficient was 0.757.

#### Religious coping scale

The religious coping scale (RCOPE) was developed by Pargament et al. in 1988 to identify positive and negative religious coping models depending on the correlation between coping, religious coping, and psychological data of three groups with different life experiences [23]. Halil Ekşi adapted the religious coping scale into Turkish in 2016 [24]. It has two subscales and ten items: seven items in positive religious coping subscale (items 1, 2, 3, 4, 5, 6, 7) and three in negative religious coping subscale (items 8, 9, 10). The scale is of a four-point Likert type. The total score of the positive religious coping subscale ranges from 7 to 28 points, whereas the total score of the negative religious coping subscale ranges from 3 to 12 points. The scale has no cut-off point. Low scores from the scale indicate that religious coping style is low, while high scores indicate high. Cronbach's internal consistency coefficient of the scale was calculated as 0.91 for the positive religious coping subscale and 0.86 for the negative religious coping subscale. RCOPE reveals the relationships and interactions between life satisfaction, resilience, and hope. It was used to examine how the variables studied in the patient relatives differed based on their demographic characteristics. This study found that Cronbach's Alpha reliability coefficient was 0.768 for the positive religious coping subscale and 0.754 for the negative religious coping subscale. Positive religious coping (PRC) subscale: This dimension includes attitudes and behaviors such as a positive relationship with God, cooperation, and asking for help (e.g., When I face a problem in life, I try to be closer to God). Negative religious coping (NRC) subscale: This dimension includes attitudes and behaviors such as being punished, being alone, not being loved by God, and being abandoned (e.g., When I face a problem in life, I believe that God punishes me for the sins I have committed).

### Data analysis

The Statistical Package for the Social Sciences (SPSS) version 22 program was employed to analyze the data. Percentage, arithmetic mean, standard deviation, median, and minimum and maximum values were used as descriptive statistics. Shapiro–Wilk test for normality and Q-Q plots were used to determine whether or not the data were normally distributed. Since the data were not normally distributed, the Mann–Whitney U test was used to compare two independent groups, and the Kruskal–Wallis test was used to compare more than two independent groups. Spearman's correlation analysis was applied to determine the correlation between scale scores. The statistical significance level was accepted as *p* < 0.05.

## Results

### Socio-demographic characteristics of the participants

It was found that the mean age of the participants was 38.84 ± 11.19 years, and most of them were married. 82.3% of the participants have nuclear families, and 61.3% are female (Table 1).

**Table 1:** Socio-demographic characteristics of the participants (*n* = 147)

	**N**	**%**
**Age** ($\overline{X }$** ± ***Sd*: 38.84** ± **11.19)		
30 years and under	45	30.6
31–40 age	42	28.6
41–50 age	30	20.4
50 years and older	30	20.4
**Gender**		
Woman	90	61.3
Men	57	38.7
**Marital Status**		
Married	115	78.2
Single	32	21.8
**Family structure**		
Nuclear	121	82.3
Extended	16	10.9
Broken	10	6.8
**Education Level**		
Literate with no formal education	6	4.1
Primary School	19	12.9
Secondary School	23	15.6
High School	56	38.1
Higher Education	43	29.3
**Working Status**		
Employed	93	63.3
Unemployed	54	36.7
**Job**		
Housewife	26	17.7
Civil Servant	34	23.2
Worker	24	16.3
Retired	13	8.8
Self-Employed	32	21.8
Student	18	12.2

### The SSCRS and RCOPE scores of the participants

Their mean scores were 27.74 ± 4.39 for the spirituality and spiritual care subscale, 15.84 ± 2.60 for the religiosity subscale, 13.57 ± 2.57 for the personalized care subscale, and 57.16 ± 6.41 for the overall SSCRS (Table 2). These results show that the participants' perception levels of spirituality and spiritual care concepts are close to good. When the Religious Coping Scale scores of the participants were examined, it was found that both Positive Religious Coping levels (23.11 ± 2.34) and Negative Religious Coping levels (9.48 ± 1.47) were close to high (Table 2).

**Table 2:** The RCOPE and SSCRS scores of the participants

**Scales**	$\overline{X }$** ± *Sd***	***Med (Min–Max)***	***Available scores***
**Religious Coping Scale**			
Positive Religious Coping subscale	23.11 ± 2.34	23 (20–28)	7–28
Negative Religious Coping subscale	9.48 ± 1.47	9 (6–12)	3–12
**Spirituality and Spiritual Care Rating Scale**			
Spirituality and Spiritual Care subscale	27.74 ± 4.39	27 (18–41)	9–45
Religiosity subscale	15.84 ± 2.60	16 (8–20)	4–20
Personalized Care subscale	13.57 ± 2.57	13 (9–20)	4–20
**Total SSCRS**	57.16 ± 6.41	56 (40–74)	17–85

*RCOPE* Religious Coping Scale, *SSCRS* Spirituality and Spiritual Care Rating Scale

### Distribution of RCOPE scores of the participants in terms of some of their socio-demographic characteristics

When the RCOPE scores of the participants were examined according to some of their socio-demographic characteristics, it was determined that the variable of working status affected the positive religious coping subscale mean scores, the employed participants had lower mean scores of positive religious coping subscale, and the difference between the groups was statistically significant (*p* = 0.012). The employed participants had lower mean scores of negative religious coping subscale, and the difference between the groups was statistically significant (*p* = 0.045). Participants who lived in the same house with their patients had higher mean scores on the positive religious coping subscale than those who did not, and the difference between the groups was statistically significant (*p* = 0.018) (Table 3).

**Table 3:** Distribution of RCOPE scores of the participants in terms of some of their socio-demographic characteristics

Characteristics	*n*	SCOPE	
		**Positive Religious Coping** $\overline{X }$** ± *Sd***	**Negative Religious Coping** $\overline{X }$** ± *Sd***
**Age**			
30 years and under	45	22.95 ± 2.19	9.37 ± 1.36
		23 (20–28)	9 (6–12)
31–40 years	42	22.85 ± 1.99	9.50 ± 1.38
		22 (20–27)	9 (7–12)
41–50 years	30	23.60 ± 2.63	9.63 ± 1.73
		23.5 (20–27)	9 (6–12)
50 years and older	30	23.23 ± 2.71	9.50 ± 1.52
		22 (20–28)	9.5 (6–12)
***Test***^***a***^	***KW***	*1.058*	*0.769*
	***p***	*0.787*	*0.857*
**Marital status**			
Married	115	23.13 ± 2.32	9.49 ± 1.45
		23 (20–28)	9 (6–12)
Single	32	23.03 ± 2.42	9.46 ± 1.56
		23 (20–28)	9 (7–12)
***Test***^***a***^	***z***	*-0.294*	*-0.277*
	***p***	*0.768*	*0.782*
**Employment Status**			
Employed	93	22.69 ± 2.07	9.30 ± 1.35
		22 (20–28)	9 (6–12)
Unemployed	54	23.83 ± 2.61	9.81 ± 1.61
		23.5 (20–28)	10 (6–12)
***Test***^***b***^	***z***	***-2.522***	***-2.000***
	***p***	***0.012***	***0.045***
**Educational Level**			
Literate	6	23.16 ± 2.22	11.33 ± 1.03a
		23 (20–26)	12 (10–12)
Primary school	19	24.63 ± 2.87	9.36 ± 1.53b
		26 (20–28)	9 (6–12)
Secondary school	23	22.95 ± 2.18	9.08 ± 1.56b
		23 (20–27)	9 (7–12)
High school	56	22.62 ± 2.27	9.44 ± 1.27b
		22 (20–28)	9 (6–12)
Higher education and higher	43	23.16 ± 2.06	9.55 ± 1.54b
		23 (20–27)	9 (6–12)
**Test**^**a**^	***KW*** ***p***	*8.971* *0.062*	*10.952* *0.027*
**Status of living with the patient in the same house**			
Yes	62	23.66 ± 2.39	9.45 ± 1.56
		23 (20–28)	9 (6–12)
No	85	22.72 ± 2.23	9.52 ± 1.41
		22 (20–28)	9 (6–12)
***Test***^***b***^	***z***	***-2.361***	*-0.188*
	***p***	***0.018***	*0.851*

*RCOPE* Religious Coping Scale, *SSCRS* Spirituality and Spiritual Care Rating Scale

^a^The Kruskal Wallis test was utilized

^b^The Mann–Whitney U test was utilized

### Scale scores of the participants according to the status of contracting COVID-19

In this study, it was found that relatives of patients with positive PCR test results had higher mean scores on the SSC subscale, and the difference between the groups was statistically significant (Table 4). In addition, it was found that the mean scores of the negative religious coping subscale of the relatives of terminally ill patients exposed to COVID-19 were high, and the difference between the groups was statistically significant (*p* = 0.008) (Table 4).

**Table 4:** Scale scores of the participants according to the status of contracting COVID-19

Status of contracting COVID-19	*n*	SCOPE		SSCRS			
		**Positive Religious Coping** $\overline{X }$** ± *Sd***	**Negative Religious Coping** $\overline{X }$** ± *Sd***	**Spirituality and spiritual care** $\overline{X }$** ± *Sd***	**Religiosity** $\overline{X }$** ± *Sd***	**Personalized care** $\overline{X }$** ± *Sd***	**Total** $\overline{X }$** ± *Sd***
**Scale scores of patients' relatives according to PCR results of terminal-stage patients**							
Negative	101	23.12 ± 2.41	9.34 ± 1.43	26.92 ± 4.06	15.65 ± 2.83	13.91 ± 2.68	56.49 ± 6.39
		23 (20–28)	9 (6–12)	26 (18–39)	16 (8–20)	14 (10–20)	56 (40–74)
Positive	46	23.11 ± 2.21	9.83 ± 1.52	29.57 ± 4.61	16.26 ± 1.96	12.83 ± 2.18	58.65 ± 6.29
		22.5 (20–27)	9 (7–12)	29 (22–41)	16 (11–20)	13 (9–17)	58 (46–72)
***Test***^***a***^	***z***	*-0.038*	*-1.278*	***-2.972***	*-0.781*	***-2.137***	*-1.778*
	***p***	*0.970*	*0.201*	***0.003***	*0.435*	***0.033***	*0.075*
**The status of contracting COVID-19 in the relatives of terminally ill patients**							
No	106	23.19 ± 2.34	9.26 ± 1.42	26.84 ± 3.94	15.60 ± 2.79	13.65 ± 2.70	56.09 ± 6.27
		23 (20–28)	9 (6–12)	26 (18–39)	16 (8–20)	13 (9–20)	55 (40–74)
Yes	41	22.93 ± 2.36	10.07 ± 1.46	30.10 ± 4.68	16.46 ± 1.94	13.37 ± 2.24	59.93 ± 6.03
		22 (20–27)	9 (7–12)	30 (22–41)	16 (12–20)	14 (10–19)	59 (46–72)
***Test***^***a***^	***z***	*-0.728*	***-2.657***	***-3.637***	*-1.271*	*-0.464*	***-3.370***
	***p***	*0.467*	***0.008***	** ≤ *0.001***	*0.204*	*0.643*	***0.001***

^a^ The Mann–Whitney U test was utilized

*RCOPE* Religious Coping Scale, *SSCRS* Spirituality and Spiritual Care Rating Scale

### The correlation between the RCOPE and SSCRS scores of the participants

There was no correlation between the scores of RCOPE and SSCRS subscales (*p* > 0.05). When the correlation between the RCOPE and SSCRS scores of the participants was examined, it was found that there was a strong positive correlation between the SSCRS total score and the spirituality and spiritual care subscale score (r = 0.822, *p* ≤ 0.001). There was a weak positive correlation between the spirituality and spiritual care subscale score and the personalized care subscale score of the participants (r = 0.220, *p* = 0.007) (Table 5).

**Table 5:** The correlation between the RCOPE and SSCRS scores of the participants

Scales		PRC	NRC	SSC	R	IC	SSCRS
**PRC**	***r***	-					
	***p***						
**NRC**	***r***	0.226^**^					
	***p***	**0.006**					
**SSC**	***r***	-0.157	0.132				
	***p***	0.058	0.110				
**R**	***r***	0.012	0.156	0.154			
	***p***	0.887	0.058	0.062			
**PC**	***r***	-0.095	-0.124	0.220^**^	-0.106		
	***p***	0.255	0.133	**0.007**	0.202		
**SSCRS**	***r***	-0.115	0.110	0.822^**^	0.462^**^	0.501^**^	-
	***p***	0.164	0.184	** ≤ 0.001**	** ≤ 0.001**	** ≤ 0.001**	

*PRC* Positive Religious Coping Subscale, *NRC* Negative Religious Coping Subscale, *SSC* Spirituality and Spiritual Care Subscale, *R* Religiosity subscale, *PC* Personalized Care subscale, *SSCRS* Spirituality and Spiritual Care Rating Scale

The Spearman’s Correlation Analysis was utilized ** Correlation is significant at the 0.01 level (2-tailed)

## Discussion

This study was conducted to determine the spiritual care perceptions and religious coping methods among the relatives of terminally ill patients hospitalized in the intensive care unit.

The present study's results revealed that the participants' mean score was 27.74 ± 4.39 in the SSC (Table 2). This result shows that the participants' perceptions of spirituality and spiritual care are moderate. For the items in this subscale, participants received a minimum of 18 and a maximum of 41 points (Median 27). Participants' perceptions of spirituality and spiritual care increase as the score increases. In the study conducted by Yılmaz and Okyay [25] on nurses, the mean score of spirituality and spiritual care subscale was found to be 25.10 ± 3.44, and in the study conducted by Kostak, M. A. and Çelikkalp, U. [26] on nurses and midwives in a regional hospital, it was found to be 26.20 ± 3.95. Moreover, the study by Gönenç et al. [27] reported that the mean score of the subjects was 16.54 ± 3.3315 in the SSC subscale. Considering the limited number of studies on the subject in patients' relatives, the different results in the literature may be associated with the study population's religious and cultural beliefs, spiritual values, family structure, environment, and diversity. In the present study, the participants had a mean score of 15.84 ± 2.60 in the 'religiosity subscale' of SSCRS (Table 2). Given that the highest score of the religiosity subscale is 20, it can be asserted that the participants attached importance to the religious dimension of spirituality. A study conducted by Dehghanrad et al. in 2020 with 300 patient relatives reported that while 76.7% of the subjects had a high level of spiritual health, 23.3% had a moderate level of spiritual health. They highlighted that the religious dimension of spirituality raised patients' hope, facilitated keeping them away from stress, and brought them a positive perspective [28]. The participants had a mean score of 13.57 ± 2.57 in the PC subscale of SSCRS in the present study (Table 2). Likewise, this value was found to be 11.13 ± 2.02 in the study by Yılmaz and Okyay [25]. In this study, the mean SSCRS total score of the participants (57.16 ± 6.41) was found to be high (Table 2). Likewise, Kostak, M. A., and Çelikkalp, U. reported in their study that the total mean score of SSCRS was 63.04 ± 6.82 [26]. In their study conducted in 2020, Góes and Crossetti argued that spirituality is necessary until the last period of life, and the patient should be cared for accordingly [29]. Cultural factors and religious values of the patient's relatives may affect this result. Considering that nursing is based on addressing individuals within the framework of holistic care, spirituality is one of the fundamental values that build this integrity.

Spiritual values are intangible and subjective; therefore, it is tough to recognize them. Hence, education is essential to identify spiritual care and religious coping with death [30, 31]. When the correlation between the educational level of the patient relatives and their total mean scores in the NRC subscale was analyzed, the mean scores of the negative religious coping subscale were significantly higher in literate subjects than those who graduated from any school (*p* = 0.027) (Table 3). According to this finding, it can be asserted that those who graduate from any school receive information about religion in formal education institutions. This situation may be practical in their perception of religion and using it as a coping method. Sohail examined the religious coping strategies of individuals suffering from chronic diseases in Pakistan based on their socio-demographic characteristics and found that education level was inversely correlated with religious coping [32]. The study by Kes and Yıldırım [33] on the families of patients reported no correlation between education level and attitudes towards death and religious coping. In their study, Hood et al. [34] noted that higher education level was directly correlated with spiritual experiences and religious coping. Studies are reporting different results concerning the correlation between education level and negative religious coping. These different results may stem from various factors such as the country of these studies, cultural considerations, and belief patterns.

It was determined that the participants who lived with their patients in the same house had higher mean scores in the PRC subscale, and the difference between the groups was statistically significant (*p* = 0.018) (Table 3). The participants who lived with their patients in the same house went through the process of accepting the disease together with their patients, which had a positive effect on religious coping. Likewise, a study conducted with caregiver family members of cancer patients revealed that the subscale mean scores and quality of life were low in subjects who were sharing the house with their patients compared to those who were not, except for the status of accepting the disease [35]. Family members of terminally ill patients are burdened with heavy responsibilities. The difficulties experienced by patients who live with family members in the same house include their disease-related demands, expecting help under all circumstances but not receiving such help in the home setting, sleeplessness, and feeling physically exhausted. For relatives of terminally ill patients, it is tough to know that loved ones suffer from a life-threatening illness, to monitor their hospitalization, and to cope with the concern of losing them. Therefore, attaching importance to spiritual health and religious beliefs and values is the best psychological support. Healthcare professionals should cooperate with the families and thus identify ways to help them.

Caregiver family members of terminally ill patients experience interruptions in their activities of daily living and their professional roles. Most of the sample in this study were employed (*n* = 93) (Table 1). It was determined that the variable of working status affected the positive religious coping subscale mean scores, the employed participants had lower mean scores of positive religious coping subscale, and the difference between the groups was statistically significant (*p* = 0.012). The employed participants had lower mean scores of negative religious coping subscale, and the difference between the groups was statistically significant (*p* = 0.045) (Table 3). The study conducted by Karakartal [36] with the caregiver family members of terminally ill patients during the treatment revealed that the subjects experienced financial problems since they could not steadily be employed, and they also suffered from restrictions in their social lives due to their caregiving obligations. In this study, the fact that the participants who continued their working life had lower scores in both religious coping subscales may have developed due to the stress, pressure, and time constraints brought by business life, the difficulty of balancing between spiritual needs and business needs, and the neglect of employees' spiritual and religious needs.

Fear, loneliness, isolation, and diseases people experienced as a consequence of the COVID-19 pandemic indicate that spiritual care should be prioritized by families and healthcare professionals [37]. This study found that the mean scores in the NRC subscale were higher in terminally ill patients and relatives of terminally ill patients who were found to have COVID-19 (Table 4). The study's findings suggested that stress adversely affected the religious coping strategies used by the subjects. A previous study indicated that most participants (86%) had increased religious coping practices during the COVID-19 pandemic. They started practices such as prayer, worship, and dhikr [38]. A study conducted with 57 people in Brazil found that when the subjects had an awareness of life during the COVID-19 pandemic, this strengthened their resilience, and they relieved themselves through religious coping [39]. This study found that relatives of patients with positive PCR test results had higher mean scores on the SSC subscale, and the difference between the groups was statistically significant (Table 4). This result suggested that relatives with PCR-positive patients found more consolation through spirituality. When the literature was examined, no study comparing COVID-19 with the spiritual orientation of patient relatives was found. This is because COVID-19 is a rapidly spreading disease; therefore, hospitals imposed visitor bans, patient relatives could not help their patients, they were at risk of infection, and they were exposed to social isolation during the quarantine period. Especially families with patients staying in the intensive care unit should be effectively informed and supported by healthcare professionals on spiritual care. The SSCRS total mean score was found to be higher in participants who contracted COVID-19 or had family members contracting COVID-19 (Table 4) (*p* < 0.001). Being diagnosed with COVID-19 inhibits the interaction between patients and their relatives. In this case, it can be asserted that the expectations of patient relatives, nurses, and patients for spiritual care needs increased. Different from the findings of the present study, Kasapooğlu found a significant positive correlation in the spirituality subscale applied to individuals during the COVID-19 pandemic (*p* < 0.01) and argued that spirituality helped to prevent the stress caused by the COVID-19 pandemic and enabled individuals to have new hopes and goals by bonding them with religious and spiritual values; thus, resulting in keeping them resilient [40]. In the study by Kaplan, Sevinç, and İşbilen., [38] with 3597 people from different segments of society during the COVID-19 pandemic, 88.2% of the participants stated they needed spiritual care. Also, 66.2% of them thought that in case of events such as epidemics or disasters, religious and spiritual care experts take charge so people can express their feelings and thoughts. Terminally ill patients who are treated in intensive care and palliative care clinics and are diagnosed with COVID-19 are the group of patients and relatives most in need of spiritual care and religious coping with illness [37, 41]. Although there are studies on such patients in Turkey, the number of studies on patient relatives is limited.

No significant correlation was found between the SSCRS total score and positive and negative religious coping subscale scores. However, when comparing the SSCRS total score and the score of the SSC subscale, a strong positive correlation was found between them (Table 5) (*p* ≤ 0.001). As the SSCRS scores increased, the subscale scores also significantly increased. It can be asserted that the patient's relatives were sensitive to attributing meaning to spirituality and attaching importance to spiritual care. Spirituality is a notion that encompasses an individual’s values, beliefs, life experiences, and the pursuit of meaning in life [42]. In the study conducted by Sülü Uğurlu and Başbakkal to determine the needs of patient relatives for spiritual support, they reported that the subjects adopted and approached spirituality from a positive perspective, felt more resilient, and trusted the medical interventions [43]. Dağ and Badır conducted a study with nurses working in intensive care units. They reported that 78.4% of nurses stated that involving family members in the care of terminally ill patients is essential and that religious and spiritual duties should be fulfilled [44].

In their study on delivering spiritual care by nurses in 2019, Harrad et al. found that patients recovered faster due to meeting spiritual needs [45]. They observed that nurses had an essential role in delivering spiritual care. A quantitative study conducted with the participation of 200 nurses revealed that nurses who were trained in spiritual care had a higher quality of care than nurses who had clinical experience but were not trained in spiritual care. The spirituality and spiritual care subscale includes allocating time for spiritual care, alleviating the anxiety of the patient's relatives, empathizing with their sorrow, and respecting the cultural beliefs and religious values of family members [46–48]. These findings of the present study are essential in terms of indicating the necessity of spiritual care for terminally ill patients. Given that spirituality is a notion that covers religion, patient relatives may consider religion as a means of sanctuary.

### Limitations

Since the study was only carried out in a regional hospital's Anesthesiology and Reanimation Intensive Care Unit, its results cannot be generalized.

## Conclusion

As a result, it was determined that the level of perception of spirituality and spiritual care concepts of the relatives of terminally ill patients during the COVID-19 pandemic was close to sound, and their Positive Religious Coping levels were high. Epidemics are a reality of the world, and it is essential to learn lessons from this process and take precautions for the future. We offer a perspective to realize the coping power of religion and spirituality, which are integral parts of life.

The study observed that the patient's relatives sharing the house with their patients had high positive religious coping scores. Considering that religion is a part of spirituality and sometimes a positive coping method, it is thought that if an environment is allocated for the patient's relatives, accompanying their patients in the hospital to perform their religious rituals, then this will enhance the quality of care.

In another finding of this study, it was found that relatives of patients with positive PCR test results had higher mean scores on the spirituality and spiritual care subscale. During the COVID-19 pandemic, people have experienced intense fear of death, both for themselves and their patients. Moreover, the fear of death and the failure to accompany the patients during this period and take care of them adequately may have increased the expectations from the nurses. It will be beneficial to make plans to meet the spiritual care needs of patients and their relatives during the terminal period of infectious diseases such as COVID-19. In addition, if nurses involve relatives in caring for patients in intensive care units and examine their perceptions and expectations regarding spiritual care, this will enhance the quality of holistic care.

## Data Availability

The authors cannot make the study materials available to other researchers for reproduction. This is because they cannot share raw data due to ethical constraints. (Sharing data from individuals contradicts the "Informed Consent Statement" given to the participants by the researchers). Data used and analyzed during the current study are available from the corresponding author upon reasonable request.
